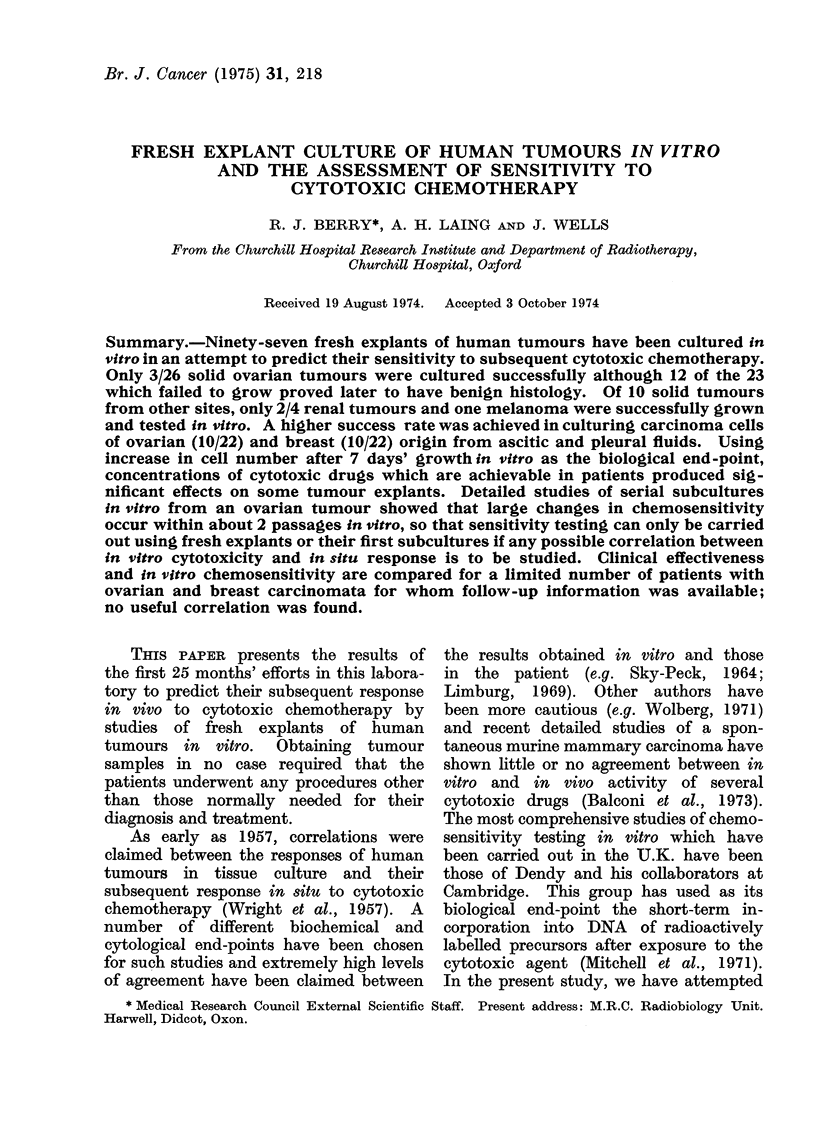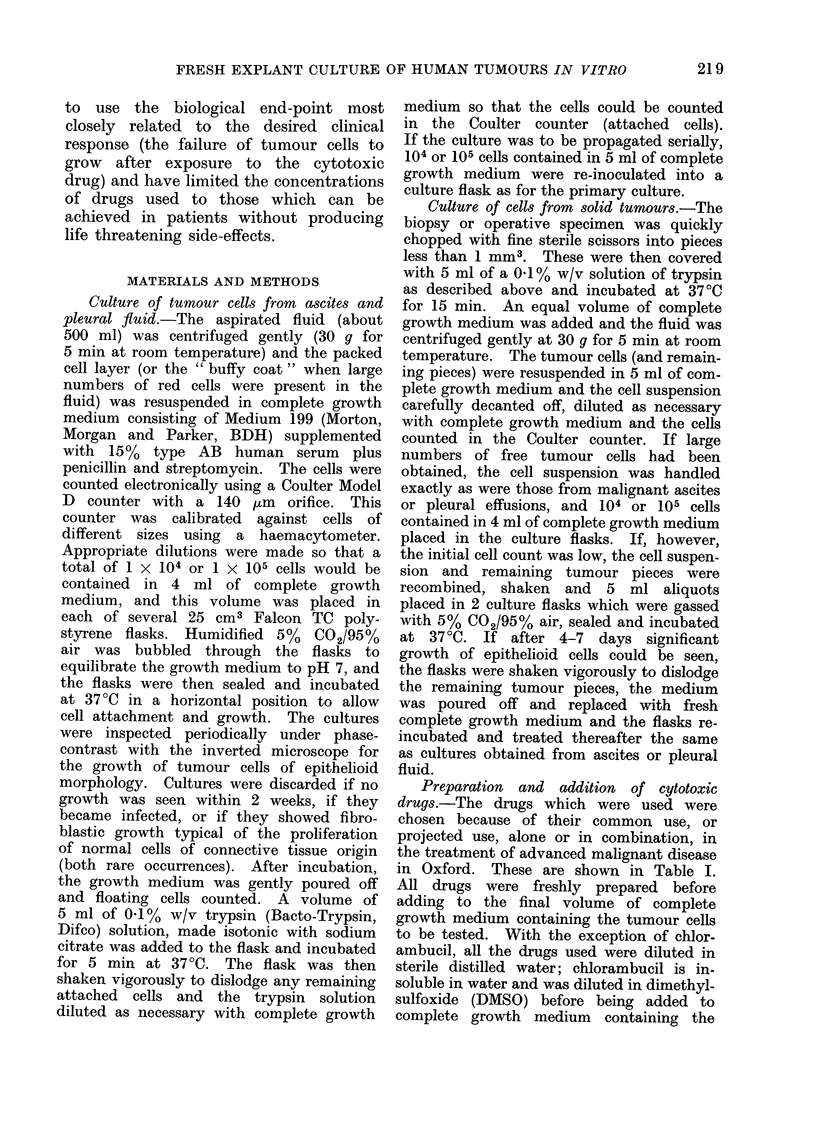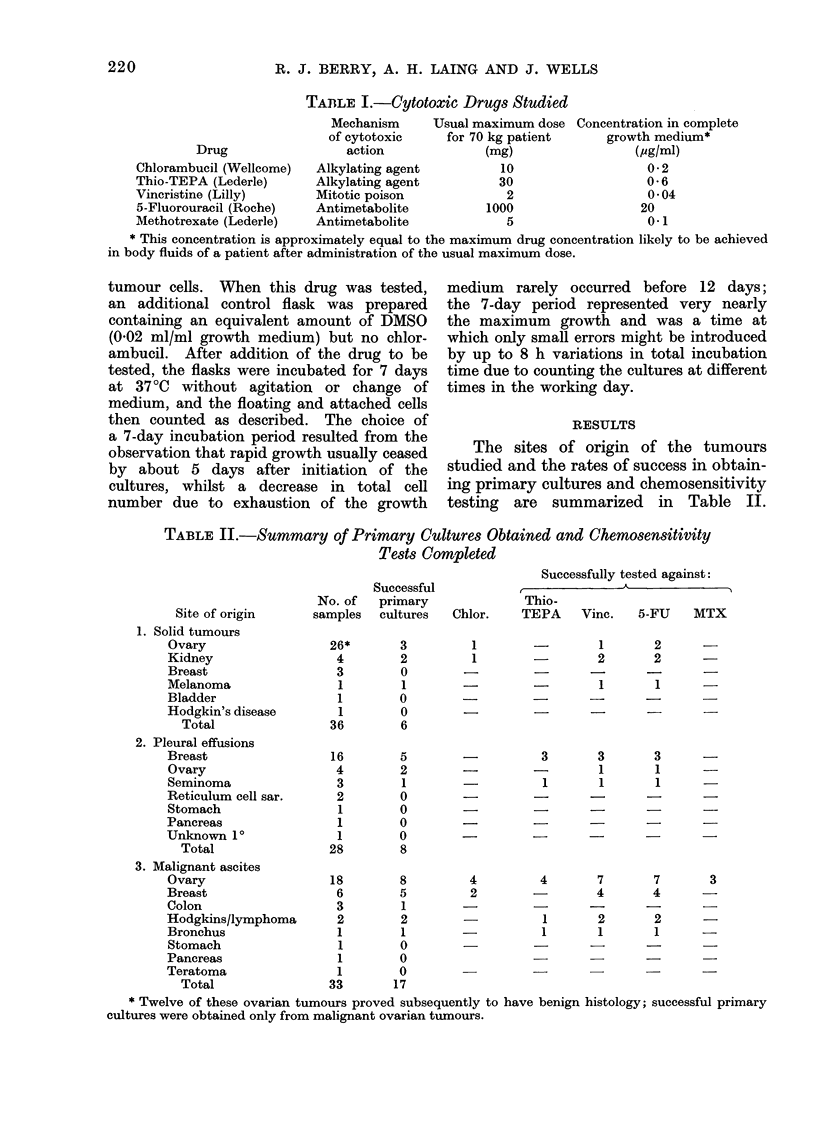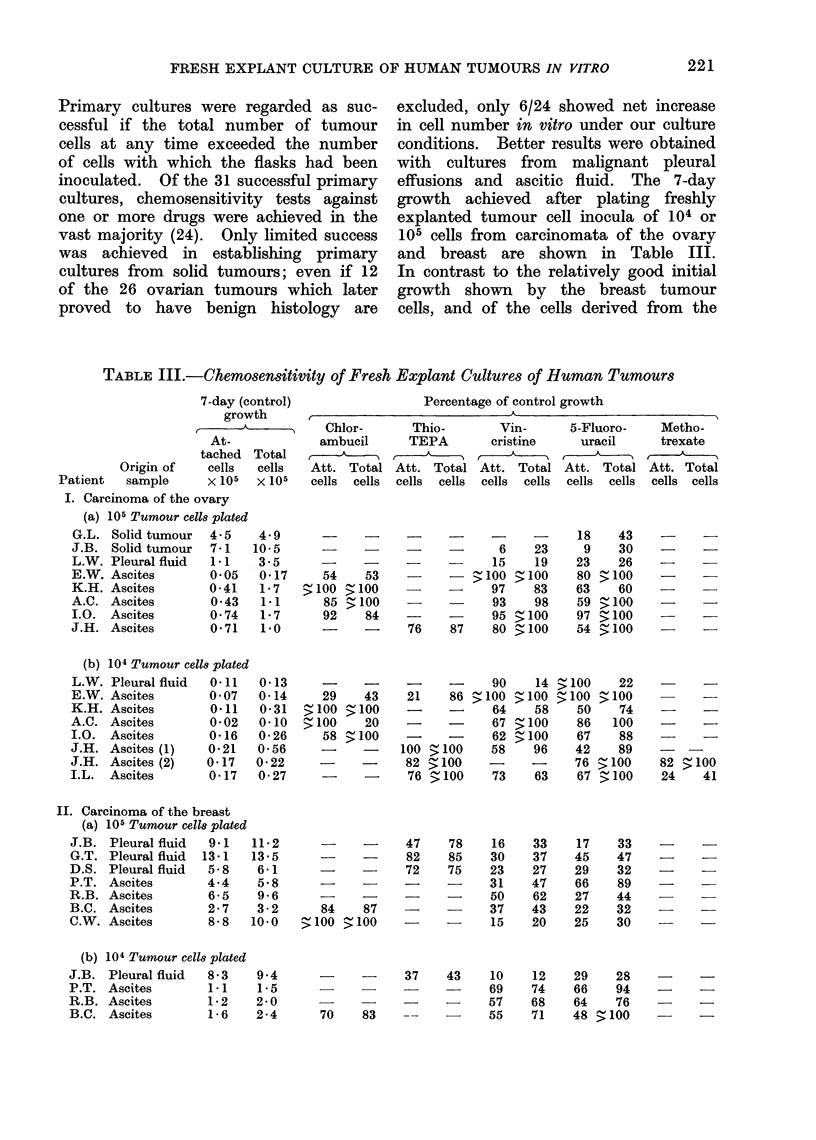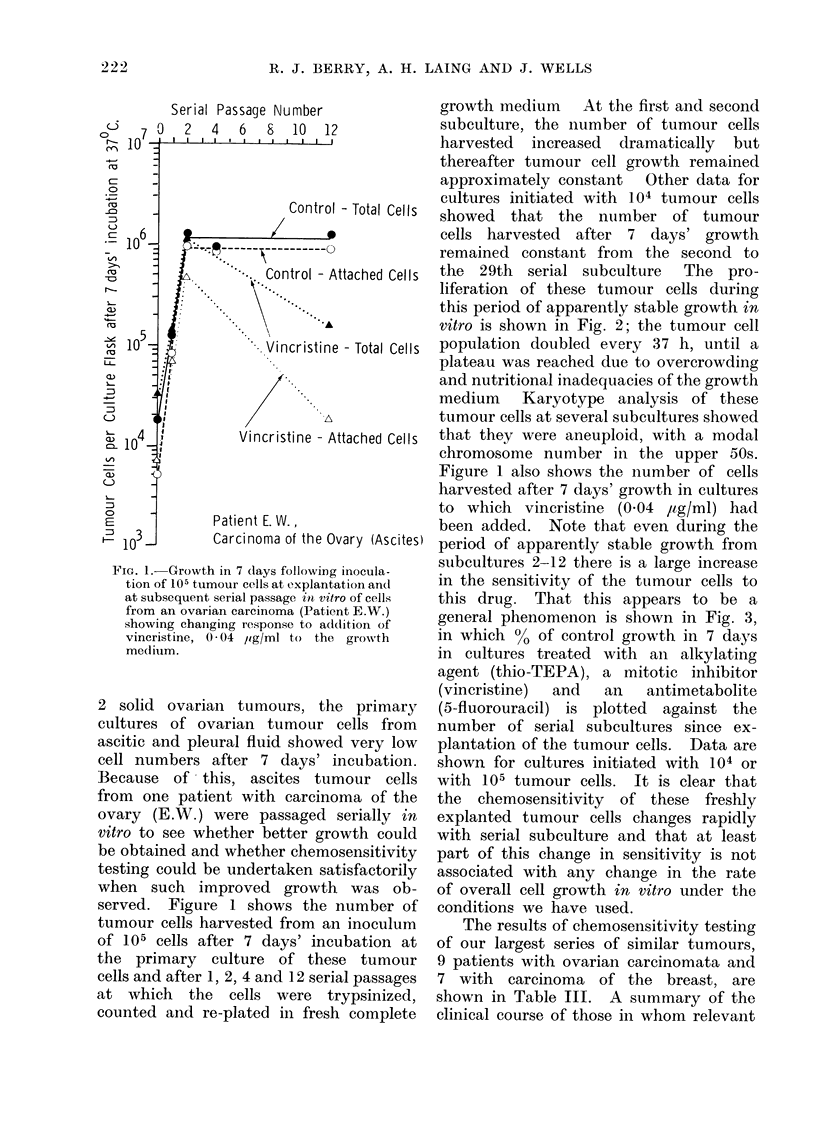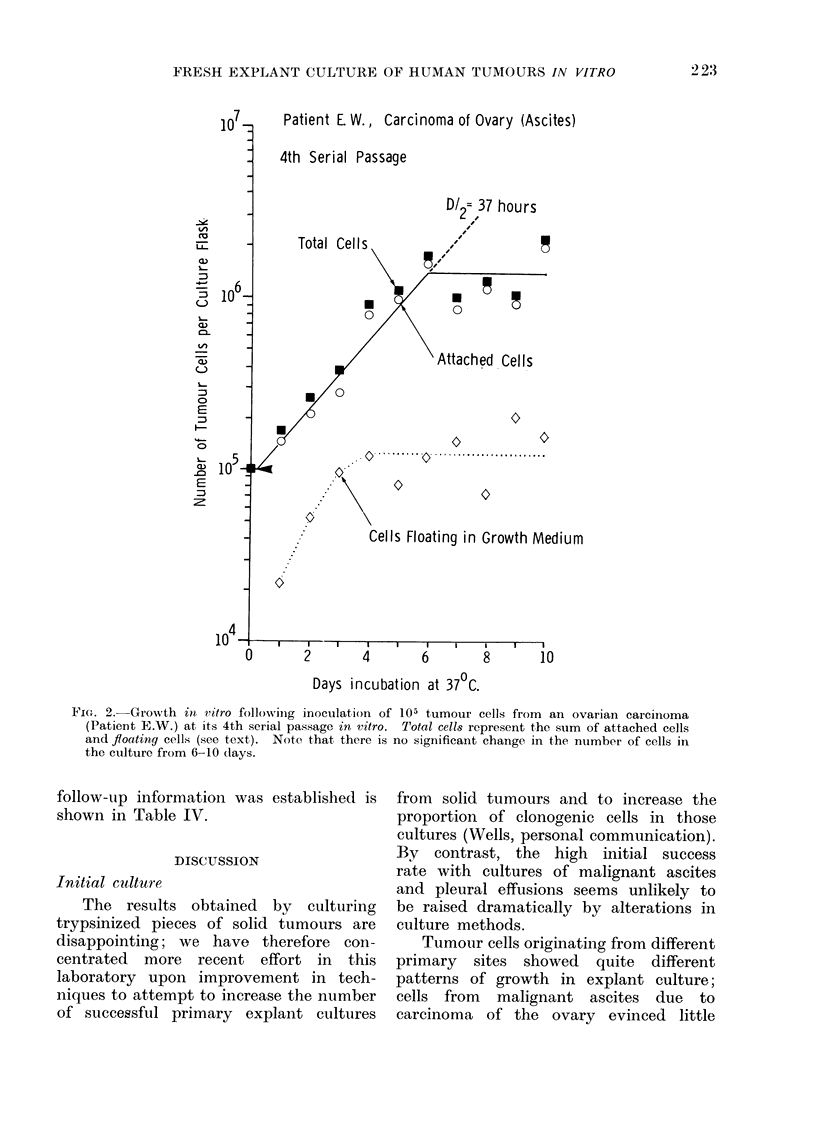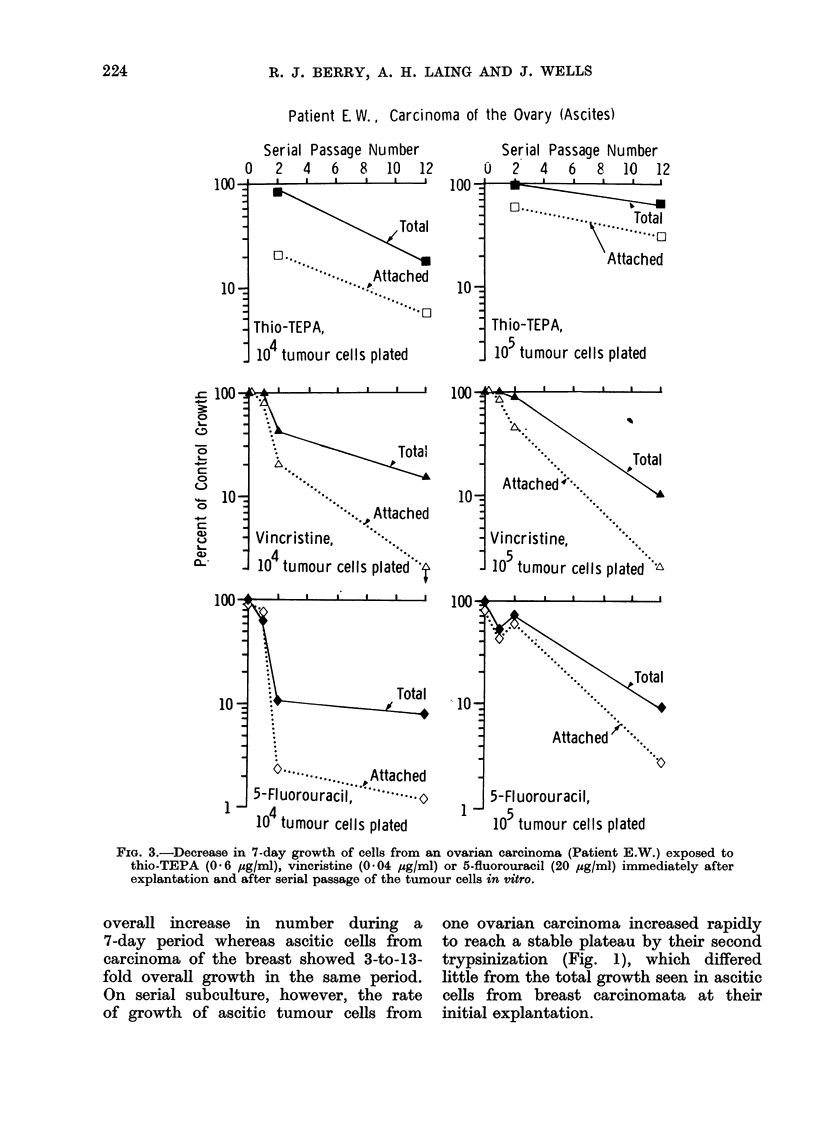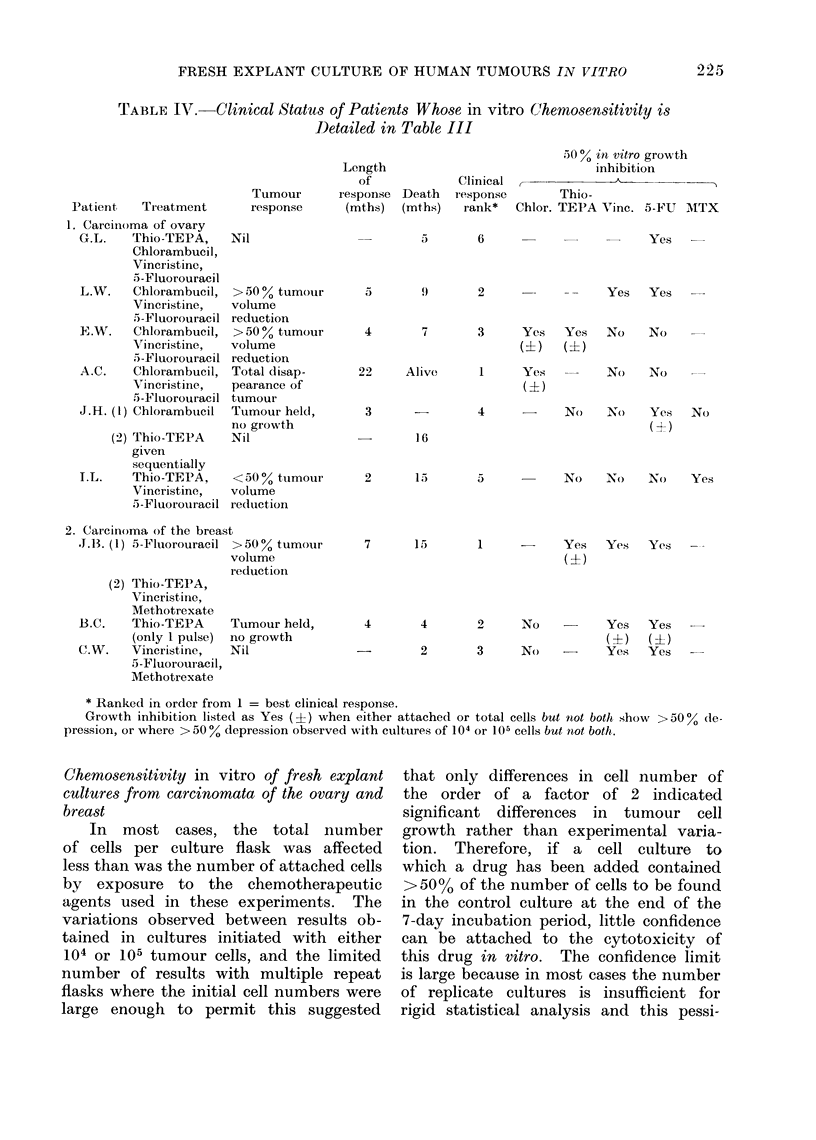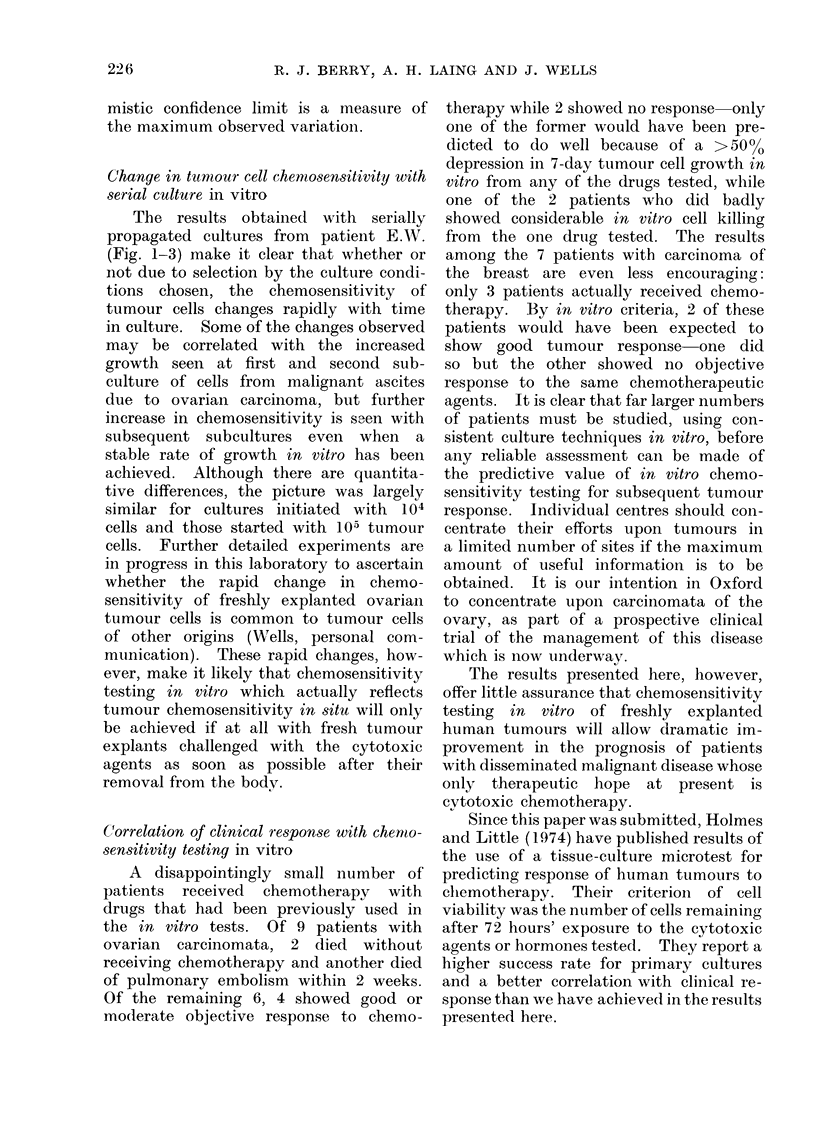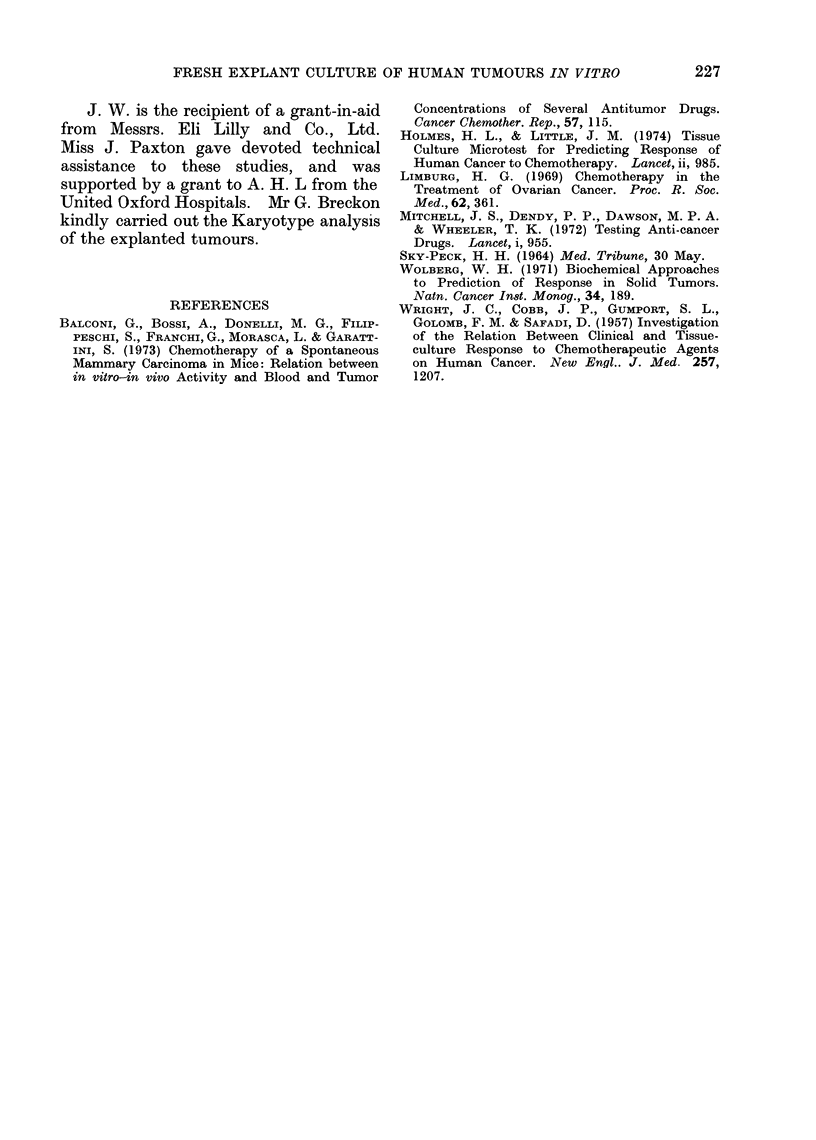# Fresh explant culture of human tumours in vitro and the assessment of sensitivity to cytotoxic chemotherapy.

**DOI:** 10.1038/bjc.1975.28

**Published:** 1975-02

**Authors:** R. J. Berry, A. H. Laing, J. Wells

## Abstract

Ninety-seven fresh explants of human tumours have been cultured in vitro in an attempt to predict their sensitivity to subsequent cytotoxic chemotherapy. Only 3/26 solid ovarian tumours were cultured successfully although 12 of the 23 which failed to grow proved later to have benign histology. Of 10 solid tumours from other sites, only 2/4 renal tumours and one melanoma were successfully grown and tested in vitro. A higher success rate was achieved in culturing carcinoma cells of ovarian (10/22) and breast (10/22) origin from ascitic and pleural fluids. Using increase in cell number after 7 days' growth in vitro as the biological end-point, concentrations of cytotoxic drugs which are achievable in patients produced significant effects on some tumour explants. Detailed studies of serial subcultures in vitro from an ovarian tumour showed that large changes in chemosensitivity occur within about 2 passages, in vitro, so that sensitivity testing can only be carried out using fresh explants or their first subcultures if any possible correlation between in vitro cytotoxicity and in situ response is to be studied. Clinical effectiveness and in vitro chemosensitivity are compared for a limited number of patients with ovarian and breast carcinomata for whom follow-up information was available; no useful correlation was found.


*Br. J. Cancer* (1975) **31**, 218

### FRESH EXPLANT CULTURE OF HUMAN TUMOURS *IN VITRO* AND THE ASSESSMENT OF SENSITIVITY TO CYTOTOXIC CHEMOTHERAPY

R. J. BERRY*, A. H. LAING AND J. WELLS

*From the Churchill Hospital Research Institute and Department of Radiotherapy, Churchill Hospital, Oxford*

Received 19 August 1974. Accepted 3 October 1974

**Summary.—Ninety-seven fresh explants of human tumours have been cultured *in vitro* in an attempt to predict their sensitivity to subsequent cytotoxic chemotherapy. Only 3/26 solid ovarian tumours were cultured successfully although 12 of the 23 which failed to grow proved later to have benign histology. Of 10 solid tumours from other sites, only 2/4 renal tumours and one melanoma were successfully grown and tested *in vitro*. A higher success rate was achieved in culturing carcinoma cells of ovarian (10/22) and breast (10/22) origin from ascitic and pleural fluids. Using increase in cell number after 7 days' growth *in vitro* as the biological end-point, concentrations of cytotoxic drugs which are achievable in patients produced significant effects on some tumour explants. Detailed studies of serial subcultures *in vitro* from an ovarian tumour showed that large changes in chemosensitivity occur within about 2 passages *in vitro*, so that sensitivity testing can only be carried out using fresh explants or their first subcultures if any possible correlation between *in vitro* cytotoxicity and *in situ* response is to be studied. Clinical effectiveness and *in vitro* chemosensitivity are compared for a limited number of patients with ovarian and breast carcinomata for whom follow-up information was available; no useful correlation was found.**

THIS PAPER presents the results of the first 25 months' efforts in this laboratory to predict their subsequent response *in vivo* to cytotoxic chemotherapy by studies of fresh explants of human tumours *in vitro*. Obtaining tumour samples in no case required that the patients underwent any procedures other than those normally needed for their diagnosis and treatment.

As early as 1957, correlations were claimed between the responses of human tumours in tissue culture and their subsequent response *in situ* to cytotoxic chemotherapy (Wright *et al.*, 1957). A number of different biochemical and cytological end-points have been chosen for such studies and extremely high levels of agreement have been claimed between the results obtained *in vitro* and those in the patient (*e.g.* Sky-Peck, 1964; Limburg, 1969). Other authors have been more cautious (*e.g.* Wolberg, 1971) and recent detailed studies of a spontaneous murine mammary carcinoma have shown little or no agreement between *in vitro* and *in vivo* activity of several cytotoxic drugs (Balconi *et al.*, 1973). The most comprehensive studies of chemosensitivity testing *in vitro* which have been carried out in the U.K. have been those of Dendy and his collaborators at Cambridge. This group has used as its biological end-point the short-term incorporation into DNA of radioactively labelled precursors after exposure to the cytotoxic agent (Mitchell *et al.*, 1971). In the present study, we have attempted

* Medical Research Council External Scientific Staff. Present address: M.R.C. Radiobiology Unit. Harwell, Didcot, Oxon.

to use the biological end-point most closely related to the desired clinical response (the failure of tumour cells to grow after exposure to the cytotoxic drug) and have limited the concentrations of drugs used to those which can be achieved in patients without producing life threatening side-effects.

### MATERIALS AND METHODS

*Culture of tumour cells from ascites and pleural fluid.*—The aspirated fluid (about 500 ml) was centrifuged gently (30 *g* for 5 min at room temperature) and the packed cell layer (or the " buffy coat " when large numbers of red cells were present in the fluid) was resuspended in complete growth medium consisting of Medium 199 (Morton, Morgan and Parker, BDH) supplemented with 15% type AB human serum plus penicillin and streptomycin. The cells were counted electronically using a Coulter Model D counter with a 140 $\mu$m orifice. This counter was calibrated against cells of different sizes using a haemacytometer. Appropriate dilutions were made so that a total of $1 \times 10^4$ or $1 \times 10^5$ cells would be contained in 4 ml of complete growth medium, and this volume was placed in each of several 25 cm$^3$ Falcon TC polystyrene flasks. Humidified 5% $CO_2$/95% air was bubbled through the flasks to equilibrate the growth medium to pH 7, and the flasks were then sealed and incubated at 37°C in a horizontal position to allow cell attachment and growth. The cultures were inspected periodically under phase-contrast with the inverted microscope for the growth of tumour cells of epithelioid morphology. Cultures were discarded if no growth was seen within 2 weeks, if they became infected, or if they showed fibroblastic growth typical of the proliferation of normal cells of connective tissue origin (both rare occurrences). After incubation, the growth medium was gently poured off and floating cells counted. A volume of 5 ml of 0·1% w/v trypsin (Bacto-Trypsin, Difco) solution, made isotonic with sodium citrate was added to the flask and incubated for 5 min at 37°C. The flask was then shaken vigorously to dislodge any remaining attached cells and the trypsin solution diluted as necessary with complete growth medium so that the cells could be counted in the Coulter counter (attached cells). If the culture was to be propagated serially, $10^4$ or $10^5$ cells contained in 5 ml of complete growth medium were re-inoculated into a culture flask as for the primary culture.

*Culture of cells from solid tumours.*—The biopsy or operative specimen was quickly chopped with fine sterile scissors into pieces less than 1 mm$^3$. These were then covered with 5 ml of a 0·1% w/v solution of trypsin as described above and incubated at 37°C for 15 min. An equal volume of complete growth medium was added and the fluid was centrifuged gently at 30 *g* for 5 min at room temperature. The tumour cells (and remaining pieces) were resuspended in 5 ml of complete growth medium and the cell suspension carefully decanted off, diluted as necessary with complete growth medium and the cells counted in the Coulter counter. If large numbers of free tumour cells had been obtained, the cell suspension was handled exactly as were those from malignant ascites or pleural effusions, and $10^4$ or $10^5$ cells contained in 4 ml of complete growth medium placed in the culture flasks. If, however, the initial cell count was low, the cell suspension and remaining tumour pieces were recombined, shaken and 5 ml aliquots placed in 2 culture flasks which were gassed with 5% $CO_2$/95% air, sealed and incubated at 37°C. If after 4–7 days significant growth of epithelioid cells could be seen, the flasks were shaken vigorously to dislodge the remaining tumour pieces, the medium was poured off and replaced with fresh complete growth medium and the flasks re-incubated and treated thereafter the same as cultures obtained from ascites or pleural fluid.

*Preparation and addition of cytotoxic drugs.*—The drugs which were used were chosen because of their common use, or projected use, alone or in combination, in the treatment of advanced malignant disease in Oxford. These are shown in Table I. All drugs were freshly prepared before adding to the final volume of complete growth medium containing the tumour cells to be tested. With the exception of chlorambucil, all the drugs used were diluted in sterile distilled water; chlorambucil is insoluble in water and was diluted in dimethylsulfoxide (DMSO) before being added to complete growth medium containing the

TABLE I.—*Cytotoxic Drugs Studied*

| Drug | Mechanism of cytotoxic action | Usual maximum dose for 70 kg patient (mg) | Concentration in complete growth medium* ($\mu$g/ml) |
|---|---|---|---|
| Chlorambucil (Wellcome) | Alkylating agent | 10 | 0·2 |
| Thio-TEPA (Lederle) | Alkylating agent | 30 | 0·6 |
| Vincristine (Lilly) | Mitotic poison | 2 | 0·04 |
| 5-Fluorouracil (Roche) | Antimetabolite | 1000 | 20 |
| Methotrexate (Lederle) | Antimetabolite | 5 | 0·1 |

* This concentration is approximately equal to the maximum drug concentration likely to be achieved in body fluids of a patient after administration of the usual maximum dose.

tumour cells. When this drug was tested, an additional control flask was prepared containing an equivalent amount of DMSO (0·02 ml/ml growth medium) but no chlorambucil. After addition of the drug to be tested, the flasks were incubated for 7 days at 37°C without agitation or change of medium, and the floating and attached cells then counted as described. The choice of a 7-day incubation period resulted from the observation that rapid growth usually ceased by about 5 days after initiation of the cultures, whilst a decrease in total cell number due to exhaustion of the growth medium rarely occurred before 12 days; the 7-day period represented very nearly the maximum growth and was a time at which only small errors might be introduced by up to 8 h variations in total incubation time due to counting the cultures at different times in the working day.

### RESULTS

The sites of origin of the tumours studied and the rates of success in obtaining primary cultures and chemosensitivity testing are summarized in Table II.

TABLE II.—*Summary of Primary Cultures Obtained and Chemosensitivity Tests Completed*

| Site of origin | No. of samples | Successful primary cultures | Successfully tested against: Chlor. | Thio-TEPA | Vinc. | 5-FU | MTX |
|---|---|---|---|---|---|---|---|
| 1. Solid tumours | | | | | | | |
| Ovary | 26* | 3 | 1 | — | 1 | 2 | — |
| Kidney | 4 | 2 | 1 | — | 2 | 2 | — |
| Breast | 3 | 0 | — | — | — | — | — |
| Melanoma | 1 | 1 | — | — | 1 | 1 | — |
| Bladder | 1 | 0 | — | — | — | — | — |
| Hodgkin's disease | 1 | 0 | — | — | — | — | — |
| Total | 36 | 6 | | | | | |
| 2. Pleural effusions | | | | | | | |
| Breast | 16 | 5 | — | 3 | 3 | 3 | — |
| Ovary | 4 | 2 | — | — | 1 | 1 | — |
| Seminoma | 3 | 1 | — | 1 | 1 | 1 | — |
| Reticulum cell sar. | 2 | 0 | — | — | — | — | — |
| Stomach | 1 | 0 | — | — | — | — | — |
| Pancreas | 1 | 0 | — | — | — | — | — |
| Unknown 1° | 1 | 0 | — | — | — | — | — |
| Total | 28 | 8 | | | | | |
| 3. Malignant ascites | | | | | | | |
| Ovary | 18 | 8 | 4 | 4 | 7 | 7 | 3 |
| Breast | 6 | 5 | 2 | — | 4 | 4 | — |
| Colon | 3 | 1 | — | — | — | — | — |
| Hodgkins/lymphoma | 2 | 2 | — | 1 | 2 | 2 | — |
| Bronchus | 1 | 1 | — | 1 | 1 | 1 | — |
| Stomach | 1 | 0 | — | — | — | — | — |
| Pancreas | 1 | 0 | | — | — | — | — |
| Teratoma | 1 | 0 | — | — | — | — | — |
| Total | 33 | 17 | | | | | |

* Twelve of these ovarian tumours proved subsequently to have benign histology; successful primary cultures were obtained only from malignant ovarian tumours.

Primary cultures were regarded as successful if the total number of tumour cells at any time exceeded the number of cells with which the flasks had been inoculated. Of the 31 successful primary cultures, chemosensitivity tests against one or more drugs were achieved in the vast majority (24). Only limited success was achieved in establishing primary cultures from solid tumours; even if 12 of the 26 ovarian tumours which later proved to have benign histology are excluded, only 6/24 showed net increase in cell number *in vitro* under our culture conditions. Better results were obtained with cultures from malignant pleural effusions and ascitic fluid. The 7-day growth achieved after plating freshly explanted tumour cell inocula of $10^4$ or $10^5$ cells from carcinomata of the ovary and breast are shown in Table III. In contrast to the relatively good initial growth shown by the breast tumour cells, and of the cells derived from the

TABLE III.—*Chemosensitivity of Fresh Explant Cultures of Human Tumours*

| | | 7-day (control) growth | | Percentage of control growth | | | | | | | | | |
|---|---|---|---|---|---|---|---|---|---|---|---|---|---|
| | | | | Chlorambucil | | Thio-TEPA | | Vincristine | | 5-Fluorouracil | | Methotrexate | |
| Patient | Origin of sample | Attached cells $\times 10^5$ | Total cells $\times 10^5$ | Att. cells | Total cells | Att. cells | Total cells | Att. cells | Total cells | Att. cells | Total cells | Att. cells | Total cells |
| I. Carcinoma of the ovary | | | | | | | | | | | | | |
| (a) $10^5$ *Tumour cells plated* | | | | | | | | | | | | | |
| G.L. | Solid tumour | 4·5 | 4·9 | — | — | — | — | — | — | 18 | 43 | — | — |
| J.B. | Solid tumour | 7·1 | 10·5 | — | — | — | — | 6 | 23 | 9 | 30 | — | — |
| L.W. | Pleural fluid | 1·1 | 3·5 | — | — | — | — | 15 | 19 | 23 | 26 | — | — |
| E.W. | Ascites | 0·05 | 0·17 | 54 | 53 | — | — | ≳100 | ≳100 | 80 | ≳100 | — | — |
| K.H. | Ascites | 0·41 | 1·7 | ≳100 | ≳100 | — | — | 97 | 83 | 63 | 60 | — | — |
| A.C. | Ascites | 0·43 | 1·1 | 85 | ≳100 | — | — | 93 | 98 | 59 | ≳100 | — | — |
| I.O. | Ascites | 0·74 | 1·7 | 92 | 84 | — | — | 95 | ≳100 | 97 | ≳100 | — | — |
| J.H. | Ascites | 0·71 | 1·0 | — | — | 76 | 87 | 80 | ≳100 | 54 | ≳100 | — | — |
| (b) $10^4$ *Tumour cells plated* | | | | | | | | | | | | | |
| L.W. | Pleural fluid | 0·11 | 0·13 | — | — | — | — | 90 | 14 | ≳100 | 22 | — | — |
| E.W. | Ascites | 0·07 | 0·14 | 29 | 43 | 21 | 86 | ≳100 | ≳100 | ≳100 | ≳100 | — | — |
| K.H. | Ascites | 0·11 | 0·31 | ≳100 | ≳100 | — | — | 64 | 58 | 50 | 74 | — | — |
| A.C. | Ascites | 0·02 | 0·10 | ≳100 | 20 | — | — | 67 | ≳100 | 86 | 100 | — | — |
| I.O. | Ascites | 0·16 | 0·26 | 58 | ≳100 | — | — | 62 | ≳100 | 67 | 88 | — | — |
| J.H. | Ascites (1) | 0·21 | 0·56 | — | — | 100 | ≳100 | 58 | 96 | 42 | 89 | — | — |
| J.H. | Ascites (2) | 0·17 | 0·22 | — | — | 82 | ≳100 | — | — | 76 | ≳100 | 82 | ≳100 |
| I.L. | Ascites | 0·17 | 0·27 | — | — | 76 | ≳100 | 73 | 63 | 67 | ≳100 | 24 | 41 |
| II. Carcinoma of the breast | | | | | | | | | | | | | |
| (a) $10^5$ *Tumour cells plated* | | | | | | | | | | | | | |
| J.B. | Pleural fluid | 9·1 | 11·2 | — | — | 47 | 78 | 16 | 33 | 17 | 33 | — | — |
| G.T. | Pleural fluid | 13·1 | 13·5 | — | — | 82 | 85 | 30 | 37 | 45 | 47 | — | — |
| D.S. | Pleural fluid | 5·8 | 6·1 | — | — | 72 | 75 | 23 | 27 | 29 | 32 | — | — |
| P.T. | Ascites | 4·4 | 5·8 | — | — | — | — | 31 | 47 | 66 | 89 | — | — |
| R.B. | Ascites | 6·5 | 9·6 | — | — | — | — | 50 | 62 | 27 | 44 | — | — |
| B.C. | Ascites | 2·7 | 3·2 | 84 | 87 | — | — | 37 | 43 | 22 | 32 | — | — |
| C.W. | Ascites | 8·8 | 10·0 | ≳100 | ≳100 | — | — | 15 | 20 | 25 | 30 | — | — |
| (b) $10^4$ *Tumour cells plated* | | | | | | | | | | | | | |
| J.B. | Pleural fluid | 8·3 | 9·4 | — | — | 37 | 43 | 10 | 12 | 29 | 28 | — | — |
| P.T. | Ascites | 1·1 | 1·5 | — | — | — | — | 69 | 74 | 66 | 94 | — | — |
| R.B. | Ascites | 1·2 | 2·0 | — | — | — | — | 57 | 68 | 64 | 76 | — | — |
| B.C. | Ascites | 1·6 | 2·4 | 70 | 83 | — | — | 55 | 71 | 48 | ≳100 | — | — |

FIG. 1.—Growth in 7 days following inoculation of $10^5$ tumour cells at explantation and at subsequent serial passage *in vitro* of cells from an ovarian carcinoma (Patient E.W.) showing changing response to addition of vincristine, 0·04 μg/ml to the growth medium.

2 solid ovarian tumours, the primary cultures of ovarian tumour cells from ascitic and pleural fluid showed very low cell numbers after 7 days' incubation. Because of this, ascites tumour cells from one patient with carcinoma of the ovary (E.W.) were passaged serially *in vitro* to see whether better growth could be obtained and whether chemosensitivity testing could be undertaken satisfactorily when such improved growth was observed. Figure 1 shows the number of tumour cells harvested from an inoculum of $10^5$ cells after 7 days' incubation at the primary culture of these tumour cells and after 1, 2, 4 and 12 serial passages at which the cells were trypsinized, counted and re-plated in fresh complete growth medium At the first and second subculture, the number of tumour cells harvested increased dramatically but thereafter tumour cell growth remained approximately constant Other data for cultures initiated with $10^4$ tumour cells showed that the number of tumour cells harvested after 7 days' growth remained constant from the second to the 29th serial subculture The proliferation of these tumour cells during this period of apparently stable growth *in vitro* is shown in Fig. 2; the tumour cell population doubled every 37 h, until a plateau was reached due to overcrowding and nutritional inadequacies of the growth medium Karyotype analysis of these tumour cells at several subcultures showed that they were aneuploid, with a modal chromosome number in the upper 50s. Figure 1 also shows the number of cells harvested after 7 days' growth in cultures to which vincristine (0·04 μg/ml) had been added. Note that even during the period of apparently stable growth from subcultures 2–12 there is a large increase in the sensitivity of the tumour cells to this drug. That this appears to be a general phenomenon is shown in Fig. 3, in which % of control growth in 7 days in cultures treated with an alkylating agent (thio-TEPA), a mitotic inhibitor (vincristine) and an antimetabolite (5-fluorouracil) is plotted against the number of serial subcultures since explantation of the tumour cells. Data are shown for cultures initiated with $10^4$ or with $10^5$ tumour cells. It is clear that the chemosensitivity of these freshly explanted tumour cells changes rapidly with serial subculture and that at least part of this change in sensitivity is not associated with any change in the rate of overall cell growth *in vitro* under the conditions we have used.

The results of chemosensitivity testing of our largest series of similar tumours, 9 patients with ovarian carcinomata and 7 with carcinoma of the breast, are shown in Table III. A summary of the clinical course of those in whom relevant

FIG. 2.—Growth *in vitro* following inoculation of $10^5$ tumour cells from an ovarian carcinoma (Patient E.W.) at its 4th serial passage *in vitro*. *Total cells* represent the sum of attached cells and *floating* cells (see text). Note that there is no significant change in the number of cells in the culture from 6–10 days.

follow-up information was established is shown in Table IV.

### DISCUSSION

### *Initial culture*

The results obtained by culturing trypsinized pieces of solid tumours are disappointing; we have therefore concentrated more recent effort in this laboratory upon improvement in techniques to attempt to increase the number of successful primary explant cultures from solid tumours and to increase the proportion of clonogenic cells in those cultures (Wells, personal communication). By contrast, the high initial success rate with cultures of malignant ascites and pleural effusions seems unlikely to be raised dramatically by alterations in culture methods.

Tumour cells originating from different primary sites showed quite different patterns of growth in explant culture; cells from malignant ascites due to carcinoma of the ovary evinced little

FIG. 3.—Decrease in 7-day growth of cells from an ovarian carcinoma (Patient E.W.) exposed to thio-TEPA (0·6 μg/ml), vincristine (0·04 μg/ml) or 5-fluorouracil (20 μg/ml) immediately after explantation and after serial passage of the tumour cells *in vitro*.

overall increase in number during a 7-day period whereas ascitic cells from carcinoma of the breast showed 3-to-13-fold overall growth in the same period. On serial subculture, however, the rate of growth of ascitic tumour cells from one ovarian carcinoma increased rapidly to reach a stable plateau by their second trypsinization (Fig. 1), which differed little from the total growth seen in ascitic cells from breast carcinomata at their initial explantation.

TABLE IV.—*Clinical Status of Patients Whose* in vitro *Chemosensitivity is Detailed in Table III*

| Patient | Treatment | Tumour response | Length of response (mths) | Death (mths) | Clinical response rank* | 50% *in vitro* growth inhibition: Chlor. | Thio-TEPA | Vinc. | 5-FU | MTX |
|---|---|---|---|---|---|---|---|---|---|---|
| 1. Carcinoma of ovary | | | | | | | | | | |
| G.L. | Thio-TEPA, Chlorambucil, Vincristine, 5-Fluorouracil | Nil | — | 5 | 6 | — | — | — | Yes | — |
| L.W. | Chlorambucil, Vincristine, 5-Fluorouracil | >50% tumour volume reduction | 5 | 9 | 2 | — | — | Yes | Yes | — |
| E.W. | Chlorambucil, Vincristine, 5-Fluorouracil | >50% tumour volume reduction | 4 | 7 | 3 | Yes (±) | Yes (±) | No | No | — |
| A.C. | Chlorambucil, Vincristine, 5-Fluorouracil | Total disappearance of tumour | 22 | Alive | 1 | Yes (±) | — | No | No | — |
| J.H. (1) | Chlorambucil | Tumour held, no growth | 3 | — | 4 | — | No | No | Yes (±) | No |
| (2) | Thio-TEPA given sequentially | Nil | — | 16 | | | | | | |
| I.L. | Thio-TEPA, Vincristine, 5-Fluorouracil | <50% tumour volume reduction | 2 | 15 | 5 | — | No | No | No | Yes |
| 2. Carcinoma of the breast | | | | | | | | | | |
| J.B. (1) | 5-Fluorouracil | >50% tumour volume reduction | 7 | 15 | 1 | — | Yes (±) | Yes | Yes | — |
| (2) | Thio-TEPA, Vincristine, Methotrexate | | | | | | | | | |
| B.C. | Thio-TEPA (only 1 pulse) | Tumour held, no growth | 4 | 4 | 2 | No | — | Yes (±) | Yes (±) | — |
| C.W. | Vincristine, 5-Fluorouracil, Methotrexate | Nil | — | 2 | 3 | No | — | Yes | Yes | — |

* Ranked in order from 1 = best clinical response.

Growth inhibition listed as Yes (±) when either attached or total cells *but not both* show >50% depression, or where >50% depression observed with cultures of $10^4$ or $10^5$ cells *but not both.*

*Chemosensitivity* in vitro *of fresh explant cultures from carcinomata of the ovary and breast*

In most cases, the total number of cells per culture flask was affected less than was the number of attached cells by exposure to the chemotherapeutic agents used in these experiments. The variations observed between results obtained in cultures initiated with either $10^4$ or $10^5$ tumour cells, and the limited number of results with multiple repeat flasks where the initial cell numbers were large enough to permit this suggested that only differences in cell number of the order of a factor of 2 indicated significant differences in tumour cell growth rather than experimental variation. Therefore, if a cell culture to which a drug has been added contained >50% of the number of cells to be found in the control culture at the end of the 7-day incubation period, little confidence can be attached to the cytotoxicity of this drug *in vitro*. The confidence limit is large because in most cases the number of replicate cultures is insufficient for rigid statistical analysis and this pessimistic confidence limit is a measure of the maximum observed variation.

*Change in tumour cell chemosensitivity with serial culture* in vitro

The results obtained with serially propagated cultures from patient E.W. (Fig. 1–3) make it clear that whether or not due to selection by the culture conditions chosen, the chemosensitivity of tumour cells changes rapidly with time in culture. Some of the changes observed may be correlated with the increased growth seen at first and second subculture of cells from malignant ascites due to ovarian carcinoma, but further increase in chemosensitivity is seen with subsequent subcultures even when a stable rate of growth *in vitro* has been achieved. Although there are quantitative differences, the picture was largely similar for cultures initiated with $10^4$ cells and those started with $10^5$ tumour cells. Further detailed experiments are in progress in this laboratory to ascertain whether the rapid change in chemosensitivity of freshly explanted ovarian tumour cells is common to tumour cells of other origins (Wells, personal communication). These rapid changes, however, make it likely that chemosensitivity testing *in vitro* which actually reflects tumour chemosensitivity *in situ* will only be achieved if at all with fresh tumour explants challenged with the cytotoxic agents as soon as possible after their removal from the body.

*Correlation of clinical response with chemosensitivity testing* in vitro

A disappointingly small number of patients received chemotherapy with drugs that had been previously used in the *in vitro* tests. Of 9 patients with ovarian carcinomata, 2 died without receiving chemotherapy and another died of pulmonary embolism within 2 weeks. Of the remaining 6, 4 showed good or moderate objective response to chemotherapy while 2 showed no response—only one of the former would have been predicted to do well because of a $>50\%$ depression in 7-day tumour cell growth *in vitro* from any of the drugs tested, while one of the 2 patients who did badly showed considerable *in vitro* cell killing from the one drug tested. The results among the 7 patients with carcinoma of the breast are even less encouraging: only 3 patients actually received chemotherapy. By *in vitro* criteria, 2 of these patients would have been expected to show good tumour response—one did so but the other showed no objective response to the same chemotherapeutic agents. It is clear that far larger numbers of patients must be studied, using consistent culture techniques *in vitro*, before any reliable assessment can be made of the predictive value of *in vitro* chemosensitivity testing for subsequent tumour response. Individual centres should concentrate their efforts upon tumours in a limited number of sites if the maximum amount of useful information is to be obtained. It is our intention in Oxford to concentrate upon carcinomata of the ovary, as part of a prospective clinical trial of the management of this disease which is now underway.

The results presented here, however, offer little assurance that chemosensitivity testing *in vitro* of freshly explanted human tumours will allow dramatic improvement in the prognosis of patients with disseminated malignant disease whose only therapeutic hope at present is cytotoxic chemotherapy.

Since this paper was submitted, Holmes and Little (1974) have published results of the use of a tissue-culture microtest for predicting response of human tumours to chemotherapy. Their criterion of cell viability was the number of cells remaining after 72 hours' exposure to the cytotoxic agents or hormones tested. They report a higher success rate for primary cultures and a better correlation with clinical response than we have achieved in the results presented here.

J. W. is the recipient of a grant-in-aid from Messrs. Eli Lilly and Co., Ltd. Miss J. Paxton gave devoted technical assistance to these studies, and was supported by a grant to A. H. L from the United Oxford Hospitals. Mr G. Breckon kindly carried out the Karyotype analysis of the explanted tumours.

### REFERENCES

BALCONI, G., BOSSI, A., DONELLI, M. G., FILIPPESCHI, S., FRANCHI, G., MORASCA, L. & GARATTINI, S. (1973) Chemotherapy of a Spontaneous Mammary Carcinoma in Mice: Relation between *in vitro–in vivo* Activity and Blood and Tumor Concentrations of Several Antitumor Drugs. *Cancer Chemother. Rep.*, **57**, 115.

HOLMES, H. L., & LITTLE, J. M. (1974) Tissue Culture Microtest for Predicting Response of Human Cancer to Chemotherapy. *Lancet*, ii, 985.

LIMBURG, H. G. (1969) Chemotherapy in the Treatment of Ovarian Cancer. *Proc. R. Soc. Med.*, **62**, 361.

MITCHELL, J. S., DENDY, P. P., DAWSON, M. P. A. & WHEELER, T. K. (1972) Testing Anti-cancer Drugs. *Lancet*, i, 955.

SKY-PECK, H. H. (1964) *Med. Tribune*, 30 May.

WOLBERG, W. H. (1971) Biochemical Approaches to Prediction of Response in Solid Tumors. *Natn. Cancer Inst. Monog.*, **34**, 189.

WRIGHT, J. C., COBB, J. P., GUMPORT, S. L., GOLOMB, F. M. & SAFADI, D. (1957) Investigation of the Relation Between Clinical and Tissue-culture Response to Chemotherapeutic Agents on Human Cancer. *New Engl.. J. Med.* **257**, 1207.